# ABC transporter-dependent brain uptake of the 5-HT_1B_ receptor radioligand [^11^C]AZ10419369: a comparative PET study in mouse, rat, and guinea pig

**DOI:** 10.1186/s13550-014-0064-0

**Published:** 2014-11-30

**Authors:** Miklós Tóth, Jenny Häggkvist, Andrea Varrone, Sjoerd J Finnema, Janine Doorduin, Masaki Tokunaga, Makoto Higuchi, Balázs Gulyás, Christer Halldin

**Affiliations:** Centre for Psychiatry Research, Department of Clinical Neuroscience, Karolinska Institutet, 171 76 Stockholm, Sweden; Department of Nuclear Medicine and Molecular Imaging, University Medical Center Groningen, University of Groningen, PO Box 30.001, 9700 RB Groningen, The Netherlands; Molecular Imaging Center, National Institute of Radiological Sciences, 4-9-1, Anagawa, Inage-ku Chiba, 263-8555 Japan; Imperial College - NTU, Lee Kong Chian School of Medicine, Nanyang Technological University, 639798 Singapore, Singapore

**Keywords:** Positron emission tomography (PET), [^11^C]AZ10419369, Serotonin 1B (5-HT_1B_) receptor, Cyclosporin A (CsA), Blood-brain barrier (BBB), ATP-binding cassette transporters (ABC transporters), Mouse, Rat, Guinea pig, Species difference

## Abstract

**Background:**

We have explored the possibility that the serotonin 1B receptor radioligand [^11^C]AZ10419369 is a substrate for adenosine triphosphate (ATP)-binding cassette (ABC) transporters, such as P-glycoprotein (P-gp), Mrp4, and Bcrp, in rodents and whether there is a species difference regarding its blood-brain barrier (BBB) penetration.

**Methods:**

In a series of preclinical positron emission tomography measurements, we have administered [^11^C]AZ10419369 to mice, rats, and guinea pigs under baseline conditions and, on separate experimental days, after administration of the ABC transporter inhibitor, cyclosporin A (CsA).

**Results:**

During baseline conditions, the brain uptake was low in mice and rats, but not in guinea pigs. After CsA pretreatment, the peak whole brain uptake values of [^11^C]AZ10419369 increased by 207% in mice, 94% in rats, and 157% in guinea pigs. Binding potentials (BP_ND_) could not be estimated during baseline conditions in mice and rats. After CsA pretreatment, the highest BP_ND_ values were obtained in the striatum and thalamus (BP_ND_ ≈ 0.4) in mice, while in rats, the highest binding areas were the striatum, thalamus, hypothalamus, and periaqueductal gray (BP_ND_ ≈ 0.5). In guinea pigs, we did not find any significant changes in BP_ND_ between baseline and CsA pretreatment, except in the striatum.

**Conclusions:**

The results indicate that BBB penetration of [^11^C]AZ10419369 was hindered by ABC transporter activity in mouse, rat, and guinea pig. This study highlights the importance of ABC transporters in the design of preclinical positron emission tomography (PET) studies.

**Electronic supplementary material:**

The online version of this article (doi:10.1186/s13550-014-0064-0) contains supplementary material, which is available to authorized users.

## Background

In recent years, there has been a greater emphasis placed on the use of small animal disease models during the early phases of novel drug and diagnostic biomarker development in molecular imaging with positron emission tomography (PET). However, an increasing body of evidence indicates that small animal models should be used with care [1]. The metabolic, pharmacokinetic, and toxicological data obtained in small animals should be extrapolated to other species or to humans rather carefully. Species differences may result in significant differences between the efficacy and, consequently, the safety of the same drug in the various species. These differences may stem from various factors, including species differences in drug metabolism, resulting from the genetic variations in drug-metabolizing enzymes, which in turn may alter pharmacokinetics, drug clearance, drug efficacy, and safety [2]. Another possible factor resulting in species differences is related to the differences in the transport of drug molecules (i) in the periphery in organs such as the liver, kidney, and gastrointestinal tract or (ii) in the central nervous system across the blood-brain barrier (BBB). Differences in BBB penetration can have a serious impact on the usefulness and predictive value of small animal models in the drug development process [3].

The transport of molecules across the BBB is partly passive (diffusion, based on concentration gradient differences) and is partly active (based on various transporter molecules and mechanisms) [4]. The most important transporters belong to the adenosine triphosphate (ATP)-binding cassette (ABC) superfamily. In the brain, their role is distinctive in the transport across the BBB. In the human genome, 48 genes encode the ABC transporters, which, based on sequence homologies, are divided into seven subfamilies (designated ABCA-ABCG) [5,6]. Several transporters from the ABC family are expressed in the capillaries of the rat brain. ABCB1, ABCC4, and ABCG2 are also present, but out of these, the ABCB1 subfamily encoding P-glycoproteins (P-gp; also called multidrug resistance 1 (Mdr1a)) is the most abundant. In rats, the expression of P-gp is approximately 4 times higher than that of Bcrp (ABCG2) and 12 times higher than that of Mrp4 (ABCC4) [7]. In humans, however, it has been found that Bcrp has a higher expression level than P-gp [8].

P-gp is a 170-kDa lipoprotein and is a well-characterized member of the ABC family. P-gp is widely expressed in plasma cell membranes across all vertebrate species and is upregulated in multidrug-resistant tumors [5,6]. In the BBB, P-gp contributes to the control of the pharmacokinetics of various compounds. It has been suggested that P-gp-mediated drug efflux may play a decisive role in the development and maintenance of various disorders, including, among others, multidrug-resistant cancer and drug-resistant epilepsies [9]. Differences in P-gp expression across different species have been reported for rat, guinea pig, and monkey [10].

[^11^C]AZ10419369 is an ^11^C-radiolabelled analogue of a serotonin 1B (5-HT_1B_) candidate drug, AZ10419369, and a novel radioligand developed for the selective visualization of the 5-HT_1B_ receptors with PET [11,12]. Whereas the radioligand [^11^C]AZ10419369 has been demonstrated to be a good marker of drug-induced occupancy at central 5-HT_1B_ receptors in the primate brain [13,14], initial measurements showed poor brain penetration in rats (unpublished data). In the present study, we set out to explore the possibility that [^11^C]AZ10419369 is a substrate for ABC transporters in rodents and, consequently, if there is a species difference regarding its BBB penetration.

The main objective of the present investigation was to explore the differences in BBB transport of [^11^C]AZ10419369, with special regard to the ABC transporter activities, in three rodent species widely used in preclinical drug development studies: mouse, rat, and guinea pig. PET measurements were performed after i.v. injection of [^11^C]AZ10419369 under baseline conditions as well as after pretreatment with cyclosporin A (CsA), an inhibitor of ABC transporters.

## Methods

### Animals

Eight male C57BL/6J mice, seven male Wistar rats, and seven male Dunkin-Hartley guinea pigs were used in the study. The animals were housed in groups in individually ventilated cages in a thermoregulated (approximately 22°C), humidity-controlled facility under a 12-h/12-h light/dark cycle with access to food and water *ad libitum*.

All animal experiments were conducted according to the guidelines of the Swedish National Board of Laboratory under a protocol approved by the Ethics Review Board of Northern Stockholm, Sweden (N210/10, N557/11).

### Radiochemistry and drugs

[^11^C]AZ10419369 was prepared at Karolinska Institutet by *N*-methylation of the desmethyl precursor (8-(1-piperazinyl)-5-methylchrom-2-en-4-one-2-(4-morpholi-nophenyl) carboxamide, AstraZeneca R&D, Wilmington, DE, USA), using [^11^C]methyl triflate, as described in detail earlier [11]. CsA (50 mg/mL, formulated in 96% ethanol) was purchased from Novartis Sverige AB (Täby, Sweden).

### *In vivo* imaging with PET

On the experimental day, the animals were anesthetized by inhalation of isoflurane (4% to 5% isoflurane in 100% oxygen). After induction of anesthesia, the isoflurane concentration was lowered to 1.5% to 2% (50:50 air/oxygen) and a cannula was inserted in the tail vein in mouse and rat and in the tarsal vein in the case of guinea pig. The animals were positioned in the PET camera in transaxial position with their heads in the center of the field of view. [^11^C]AZ10419369 was intravenously administered simultaneously with the start of the PET acquisition and was followed by a 0.1-mL saline flush.

Eight male C57BL/6J mice (28.3 ± 0.8 g) were intravenously injected with [^11^C]AZ10419369 twice, 1 week apart. The average injected radioactivity was 10.8 ± 0.9 MBq (*n* = 16), and the average injected mass was 0.025 ± 0.024 μg (*n* = 16). Seven male Wistar rats (322 ± 46 g) were intravenously injected with [^11^C]AZ10419369 twice, 1 week apart. The mean injected radioactivity was 21.5 ± 2.7 MBq (*n* = 14), and the average injected mass is not available. Seven male guinea pigs (514.4 ± 146.9 g) were intravenously injected with [^11^C]AZ10419369 twice, 1 week apart. The average injected radioactivity was 16.8 ± 3.2 MBq (*n* = 14), and the average injected mass was 0.020 ± 0.022 μg (*n* = 14). Upon completion of the first imaging session, the animal returned to its cage. Before the start of the second PET measurement, the animals were pretreated with 50 mg/kg CsA, administered intravenously 15 min before the injection of the radioligand through the same cannula as used for radioligand injection.

### Imaging system and reconstruction

The PET measurements were acquired using the Mediso nanoScan® PET-MRI and the nanoScan® PET-CT small animal imaging systems (Mediso Ltd., Budapest, Hungary) [15,16]. Two animals were examined at the same time in the identical PET modules of the two PET systems. The acquired list mode data was reconstructed into 25 time frames (63-min scan = 4 × 10 s, 4 × 20 s, 4 × 60 s, 7 × 180 s, 6 × 360 s) in the case of mice and rats and 35 time frames (93-min scan = 4 × 10 s, 4 × 20 s, 4 × 60 s, 7 × 180 s, 11 × 360 s) in the case of guinea pigs.

The image reconstruction was made with a fully three-dimensional maximum-likelihood expectation maximization (MLEM) algorithm with 20 iterations, without scatter and attenuation correction.

### Data analysis

The reconstructed dynamic mouse and rat PET images were co-registered to the inbuilt mouse and rat MRI templates available in PMOD, which also incorporates volume of interest (VOI) sets for mice and rats (version 3.3, PMOD Technologies Ltd., Zurich, Switzerland). Co-registration was done manually with the use of mean PET images. For the guinea pig scans, a template MRI with an in-house developed VOI set was used to co-register with the PET scans. With the help of these VOI sets, decay-corrected time activity curves (TAC) were generated. Whole brain and regional brain uptake values were expressed as percent standard uptake value (%SUV), which was calculated by normalizing regional radioactivity for injected radioactivity and body weight. Uptake values were then calculated as a summation of uptake from 10 min to the end of the PET acquisition (total 53 min in the case of rats and mice and 113 min in the case of guinea pigs).

As the %SUV is considered a semi-quantitative value, quantitative image analysis was additionally performed using PMOD. The non-displaceable binding potential (BP_ND_) was estimated using the simplified reference tissue model (SRTM) [17] using the cerebellum as reference region (mice and rats) or cerebellar gray matter (guinea pigs). This is a validated quantitative analysis method for [^11^C]AZ10419369 binding in humans [13] and has been used in non-human primates [14]. Statistical analyses were performed with Student's two-tailed, paired *t*-test.

## Results

**Figure 1:**
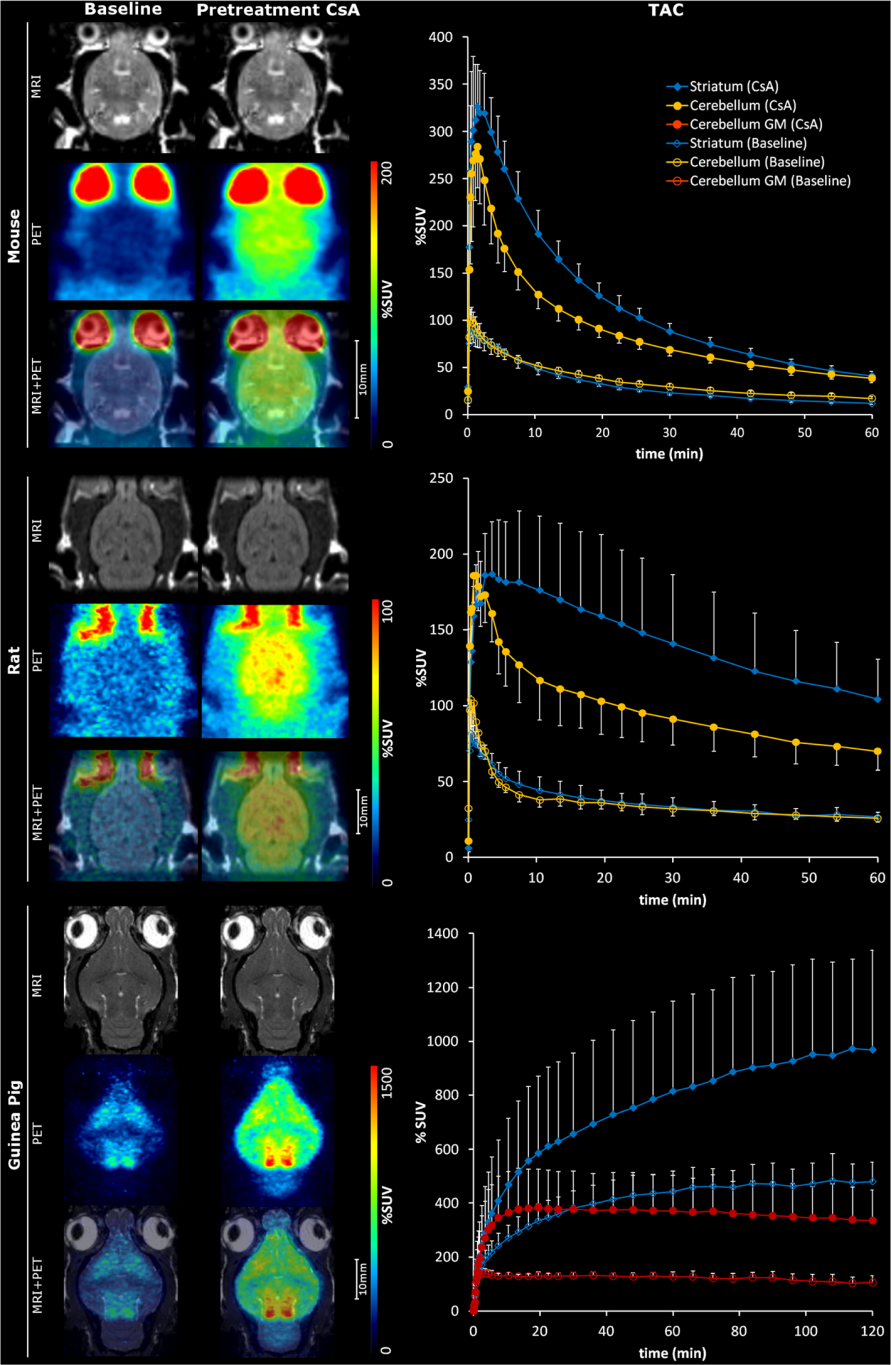
**PET images and TAC curves of [** ^**11**^ **C]AZ10419369 uptake in mouse, rat, and guinea pig.** Representative uptake images (left) and time activity curves of striatal and cerebellar uptake (right) of [^11^C]AZ10419369 in mouse (top), rat (middle), and guinea pig (bottom) in baseline and pretreatment conditions. The images are a summation from 10 min until the end of the scan (total 53 min in the case of rat and mice and 113 min in the case of guinea pig), while TAC curves are presented as mean values and SD as error bars in mice (*n* = 8), in rats (*n* = 7), and in guinea pigs (*n* = 7).

At baseline conditions, the guinea pigs showed distinguishable brain uptake compared to the surrounding tissue, whereas low brain uptake was observed in the rats and mice (Figure 1). There was a statistically significant increase in %SUV after CsA pretreatment compared to baseline images in all regions, for all animal species (Table 1). Peak whole brain uptake values increased by 207% in mice, 94% in rats, and 157% in guinea pigs after CsA pretreatment.

**Table 1:** **Comparison of regional uptake and binding potential in the different brain regions**

	**Mouse**			**Rat**			**Guinea pig**		
	**Baseline**	**CsA**	***P*** **value**	**Baseline**	**CsA**	***P*** **value**	**Baseline**	**CsA**	***P*** **value**
%SUV									
Whole brain	33.4 ± 2.4	98.6 ± 10.8	0.0000	37.4 ± 4.1	119.0 ± 29.3	0.0002	259.6 ± 34.6	643.1 ± 192.4	0.0020
Cortex	30.9 ± 2.6	95.1 ± 10.6	0.0000	38.7 ± 4.2	113.0 ± 26.7	0.0002	213.3 ± 27.3	566.9 ± 198.1	0.0027
Striatum	28.6 ± 2.4	110.4 ± 12.1	0.0000	37.0 ± 5.8	146.9 ± 43.5	0.0003	394.4 ± 58.7	768.3 ± 299.9	0.0205
Thalamus	29.3 ± 2.2	114.4 ± 14.1	0.0000	36.2 ± 5.3	145.5 ± 41.8	0.0003	301.5 ± 44.9	792.1 ± 216.4	0.0015
Hypothalamus	40.3 ± 2.3	107.8 ± 10.3	0.0000	41.2 ± 5.9	143.6 ± 39.7	0.0003	354.9 ± 60.9	794.3 ± 204.1	0.0019
Hippocampus	28.0 ± 2.2	102.6 ± 11.0	0.0000	35.6 ± 4.6	137.3 ± 40.1	0.0003	228.3 ± 31.7	605.7 ± 171.1	0.0011
Central canal-PAG	27.1 ± 1.8	106.3 ± 13.4	0.0000	37.4 ± 5.3	147.4 ± 38.7	0.0002	446.2 ± 71.8	986.5 ± 248.0	0.0027
Colliculus inferior	28.0 ± 2.6	107.1 ± 12.9	0.0000	36.7 ± 4.1	138.7 ± 38.0	0.0003	192.7 ± 30.3	900.5 ± 264.7	0.0005
Colliculus superior	28.1 ± 2.9	94.1 ± 10.2	0.0000	34.4 ± 4.3	127.9 ± 32.2	0.0002	428.4 ± 69.7	1 072.5 ± 291.8	0.0018
Midbrain	30.8 ± 2.4	111.5 ± 12.5	0.0000	37.4 ± 4.3	143.6 ± 36.7	0.0002	409.5 ± 67.6	970.1 ± 245.5	0.0018
Cerebellum	33.7 ± 3.1	81.1 ± 8.6	0.0000	34.0 ± 3.3	96.4 ± 18.3	0.0001	162.7 ± 24.8	467.5 ± 135.8	0.0006
Cerebellum GM	-	-	-	-	-	-	120.8 ± 17.4	372.6 ± 122.1	0.0009
BP_ND_									
Whole brain	-	0.19 ± 0.04	n/a	-	0.21 ± 0.10	n/a	1.56 ± 0.39	1.57 ± 0.44	n.s.
Cortex	-	0.12 ± 0.04	n/a	-	0.15 ± 0.10	n/a	1.00 ± 0.23	1.23 ± 0.30	n.s.
Striatum	-	0.39 ± 0.08	n/a	-	0.48 ± 0.21	n/a	3.13 ± 0.90	2.36 ± 0.52	0.0224
Thalamus	-	0.39 ± 0.16	n/a	-	0.48 ± 0.18	n/a	2.05 ± 0.55	2.29 ± 0.72	n.s.
Hypothalamus	-	0.30 ± 0.06	n/a	-	0.45 ± 0.15	n/a	2.76 ± 0.74	2.81 ± 1.04	n.s.
Hippocampus	-	0.25 ± 0.11	n/a	-	0.39 ± 0.18	n/a	1.25 ± 0.29	1.38 ± 0.38	n.s.
Central canal-PAG	-	0.15 ± 0.17	n/a	-	0.50 ± 0.11	n/a	4.06 ± 1.36	5.00 ± 3.04	n.s.
Colliculus inferior	-	0.17 ± 0.19	n/a	-	0.42 ± 0.12	n/a	3.08 ± 0.86	4.00 ± 1.54	n.s.
Colliculus superior	-	0.13 ± 0.06	n/a	-	0.27 ± 0.14	n/a	3.50 ± 1.15	3.65 ± 1.11	n.s.
Midbrain	-	0.26 ± 0.20	n/a	-	0.46 ± 0.11	n/a	3.34 ± 1.01	3.40 ± 1.17	n.s.
Cerebellum	-	-	-	-	-	-	0.52 ± 0.16	0.49 ± 0.44	n.s.

Regional uptake and binding potential of [^11^C]AZ10419369 in the different brain regions in mice, rats, and guinea pigs during baseline and CsA pretreatment conditions (each value represents mean ± SD). n/a, not applicable; n.s., not significant.

A fast clearance of [^11^C]AZ10419369 was demonstrated in mice and rats, while there was a slow washout in guinea pigs in both the baseline and after pretreatment with CsA (Figure 1). During baseline conditions, peak brain uptake was at 0.4 min in mice and rats and at 84 min in guinea pigs. After CsA administration, the peak brain uptake in mice was at 1.5 min and at 1.2 min in rats. However, in guinea pigs, [^11^C]AZ10419369 did not reach equilibrium during the 2-h-long PET acquisition in all regions examined besides the cerebellum.

As the initial counts in brain were very low in mice and rats at baseline condition, the binding potential of [^11^C]AZ10419369 could only be estimated reliably in guinea pigs. After administration of CsA, the brain uptake had increased sufficiently, and therefore, we quantified BP_ND_ in all three species (Table 1).

After CsA pretreatment, the highest BP_ND_ values were found in the striatum and thalamus in mice (BP_ND_ ≈ 0.4). In rats, the highest BP_ND_ values were observed in the striatum, thalamus, hypothalamus, and periaqueductal gray (BP_ND_ ≈ 0.5). In guinea pigs, the cerebellar gray matter was selected as the reference region instead of the whole cerebellum since there was a relative high concentration of [^11^C]AZ10419369 in the white matter. We found no changes in BP_ND_ between baseline and CsA pretreatment in guinea pigs except in the striatum where a significant decrease in binding (BP_ND_ 3.1 to 2.4) was observed. This is probably due to the fact that none of the regions could reach equilibrium during our experiments making the SRTM calculations unreliable despite the 2-h scan.

## Discussion

An increasing number of recent observations [10,18,19] indicate that the brain concentration of a drug can differ significantly among various species. These species differences can be traced back to various factors, including differences in plasma binding, metabolic enzyme activities, or BBB transport. As several biologically effective compounds may be substrates of efflux transporters, like P-gp, one of the most important mechanisms behind species differences in drug disposition in the brain can be traced back to this phenomenon, i.e., that a CNS drug is a substrate of P-gp in a given species.

Although CsA had been mostly used as a P-gp inhibitor in relation to the brain, in the periphery, it has been shown to inhibit transporters from the solute carrier membrane transport protein group as well [20,21]. Since brain exposure of any molecule is a function of peripheral distribution, brain penetration, and metabolism, this fact has to be taken into consideration while assessing the results. Metabolic differences under different pretreatment conditions could be a critical factor in brain uptake levels, since non-identical free fraction of [11C]AZ10419369 in the blood leads to different availability of radioligand for transport across the BBB. Arterial blood sampling is generally used in PET experiments to measure changes in the free fraction over time. This is a rather difficult task in rodents, particularly in mice, in which the required blood volume would exceed the total blood volume of the animal. The effects of blood loss from arterial blood sampling could reduce the blood volume to such extent that it may alter metabolism, influence Vt, or even cause severe health issues which could lead to exsanguination and consequently to the death of the animal.

While the effects of CsA on the periphery could have been assessed with arterial blood measurement, we did not perform this for the aforementioned reasons. These effects can be avoided by using various transporter knockout animal models. In a complementary study using Sprague-Dawley rats and Mdr1a knockout animals, we have examined the difference in brain uptake and 5-HT_1B_ receptor availability with [^11^C]AZ10419369 microPET. Increased uptake and [^11^C]AZ10419369 BP_ND_ were observed in KO rats compared with WT in some brain regions, suggesting that the brain uptake of [^11^C]AZ10419369 is influenced at least by Mdr1a function (see Additional file 1). Earlier PET studies have indicated that the brain uptake of various P-gp substrates may differ markedly between rodent and primate species [10,22-24]. It has also been demonstrated that there can be species difference in BBB transport between various rodent species [10,25]. This fact can be substantiated by the comparison of amino acid content in P-gp in rats, mice, and guinea pigs: the amino acid homology between mouse and rat is 93%, between mouse and guinea pig is 82%, and between rat and guinea pig is 80% [10].

The experiments with mice and rats showed that the rather low brain uptake of [^11^C]AZ10419369 during baseline condition could be increased significantly with the use of CsA. The brain uptake increased significantly in all species, and the binding potential became measurable after CsA pretreatment in mice and rats. In guinea pigs, the uptake of [^11^C]AZ10419369 was increased significantly, about the same amount as in mice and rats, but the BP_ND_ showed no changes compared to baseline in all regions except for the striatum, where a significant decrease was observed compared to baseline. It is possible that this difference between species could disappear with longer scanning times. However, as the guinea pigs had breathing difficulties under long-term anesthesia, increased scan time was not applied for ethical reasons.

The difference in BP_ND_ observed in guinea pigs, compared with mice and rats, is most likely due to the differences in homology of the 5-HT_1B_ receptor in the guinea pig versus that in the rat and mouse. A previous report indicated that amino acid residue 335 in the 5-HT_1B_ receptor is a major determinant of its reactivity with several ligands [26]. It has also been shown that [^3^H]AZ10419369 has high affinity (*Kd* = 0.4 nM) for both human and guinea pig 5-HT_1B_ receptors, and AZ10419369 binds to human (*Ki* = 0.8 nM) and guinea pig 5-HT_1B_ receptors (*K*_*i*_ = 0.01 nM) with higher affinity than it does to rat 5-HT_1B_ receptors (*Ki* = 2.3 nM) [12]. Furthermore, our results show good agreement with the *in vivo* results of Maier et al. (2009) on guinea pig brain distribution of [^3^H]AZ10419369 [27]. Thus, the current study seems to confirm the difference in *Kd* across species *in vivo*, with lower binding affinity to the mouse and rat 5-HT_1B_ receptor and with the highest affinity to the receptor in the guinea pig.

The results from the present study further support the importance of evaluating the outcome of small animal model experiments with caution especially when it comes to drug development studies. Since there can be species differences in transporter function, as shown in the present study, different promising radioligands targeted for the brain may be discarded on incorrect conclusions.

## Conclusions

This study showed that [^11^C]AZ10419369 is a substrate for ABC transporters in mouse, rat, and guinea pig. Our results suggest that the activity of ABC transporters, such as P-gp, as well as possible species differences in the receptor affinity of radioligands, are important factors to consider in the design of preclinical PET studies.

## Additional file

Additional file 1: **Supplementary material to the manuscript by Tóth et al.** Additional PET experiment using P-gp knockout and wild-type rats to explore the contribution of the ABC transporter P-gp on the brain uptake of [^11^C]AZ10419369.
