# Supplementary material for: ABC transporter-dependent brain uptake of the 5-HT1B receptor radioligand [11C]AZ10419369: a comparative PET study in mouse, rat, and guinea pig
Source: EJNMMI Res. 2014 Nov 30;4:64. doi: 10.1186/s13550-014-0064-0 (PMC4452686; doi:10.1186/s13550-014-0064-0)

**Supplementary material to the manuscript by Tóth et al.**

**ABC transporter dependent brain uptake of the 5-HT_1B_ receptor radioligand [^11^C]AZ10419369. A comparative pre-clinical PET study in mouse, rat and guinea pig**

The current supplementary experiments were approved by the Committee for the Care and Use of Laboratory Animals in National Institute of Radiological Sciences. P-gp knock-out (KO) rats generated by zinc finger nuclease technology were purchased from Sigma-Aldrich (St. Louis, MO, USA).

In order to explore the contribution of the ABC transporter P-gp on the brain uptake of the novel 5-HT_1B_ receptor radioligand [^11^C]AZ10419369 in the rat, we have performed a PET study using P-gp KO and wild-type rats.

Four Sprague-Dawley (389 ± 25 g) and four P-gp KO (Mdr1a-KO) (482 ± 29 g) rats were examined. The injected radioactivity was 104 ± 5.9 MBq, and the specific radioactivity was 2342 ± 189 GBq/μmol. Animals were imaged under 1.5% isoflurane anesthesia. PET measurements were made in a microPET FOCUS 220 system (Siemens Medical Solutions USA, Knoxville, TN, USA). Reconstruction was made using two-dimensional filtered back-projection with a 0.5-mm Hanning filter and the following time frames: 4 × 1, 8 × 2, and 8 × 5 min.

PET images, superimposed on an anatomical MR template, demonstrated noticeable differences in radioligand uptake between WT and P-gp KO rat brains (Figure 1).

Time-activity curves (TAC) were acquired from dynamic PET data in several rat brain regions, including the striatum and the cerebellum in WT and KO rats (Figure 2). Peak uptake of radioligand was increased by 40% in all examined areas of the P-gp KO rat brain compared to the same areas in WT animals, indicating that [^11^C]AZ10419369 is a substrate for P-gp in rats.

BP_ND_ values were estimated by the simplified reference tissue model (SRTM) with the use of the cerebellum as a reference region. In WT animals the BP_ND_ values were 0.38 ± 0.04 in striatum, 0.19 ± 0.04 in hippocampus, 0.26 ± 0.05 in cortex and 0.28 ± 0.02 in thalamus, while in the KO animals they were 0.40 ± 0.03 in the striatum, 0.27 ± 0.04 in the hippocampus, 0.27 ± 0.04 in the cortex and 0.35 ± 0.03 in the thalamus (Figure 3).

The difference in BP_ND_ between the two genotypes was not consistent among regions, and this could be attributed to inaccuracy of BP_ND_ estimation in the WT rats. BP_ND_ in P-gp KO rats might be determined with relatively high precision, but BP_ND_ values were much lower than earlier reported in primates.

The present PET measurements indicate that [^11^C]AZ10419369 is a substrate for P-gp in rats, and, accordingly, there may be a difference in binding to 5-HT_1B_ receptors between rats and primates.

**Figures**

Figure 1. – Averaged PET uptake images with [^11^C]AZ10419369 (data acquisition: 0 - 90 min) fused with MR images


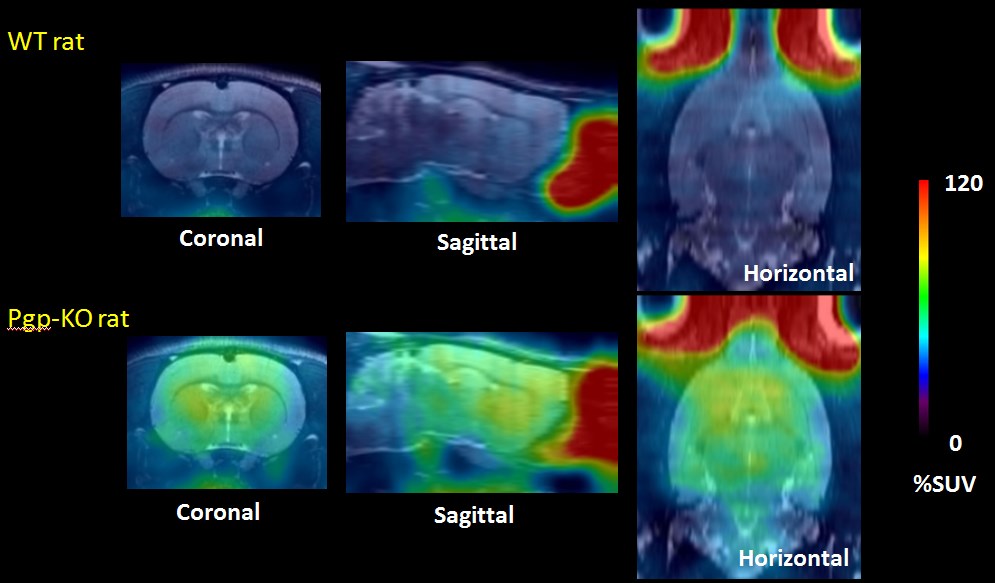


Figure 2. - TACs in the striatum and cerebellum of WT and KO rats


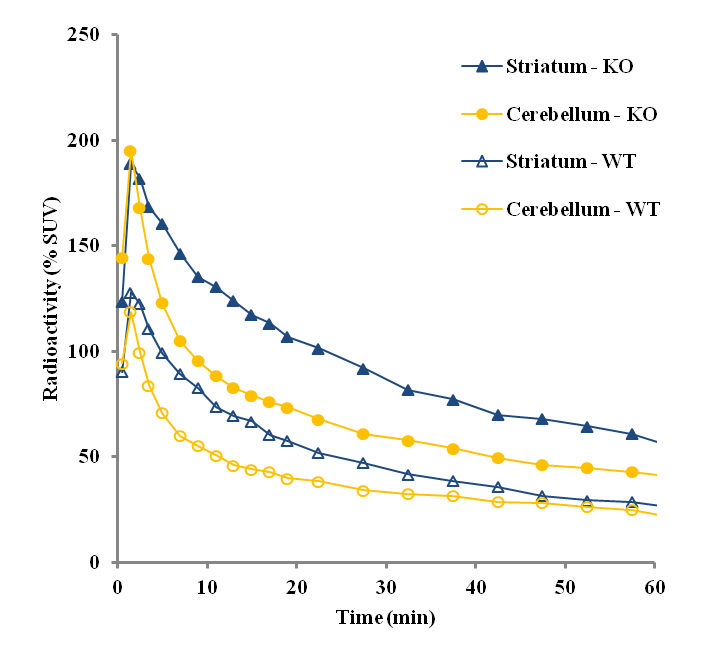


Figure 3. - BP_ND_ values in WT and P-gp KO rats estimated by analyzing in vivo PET data with SRTM.


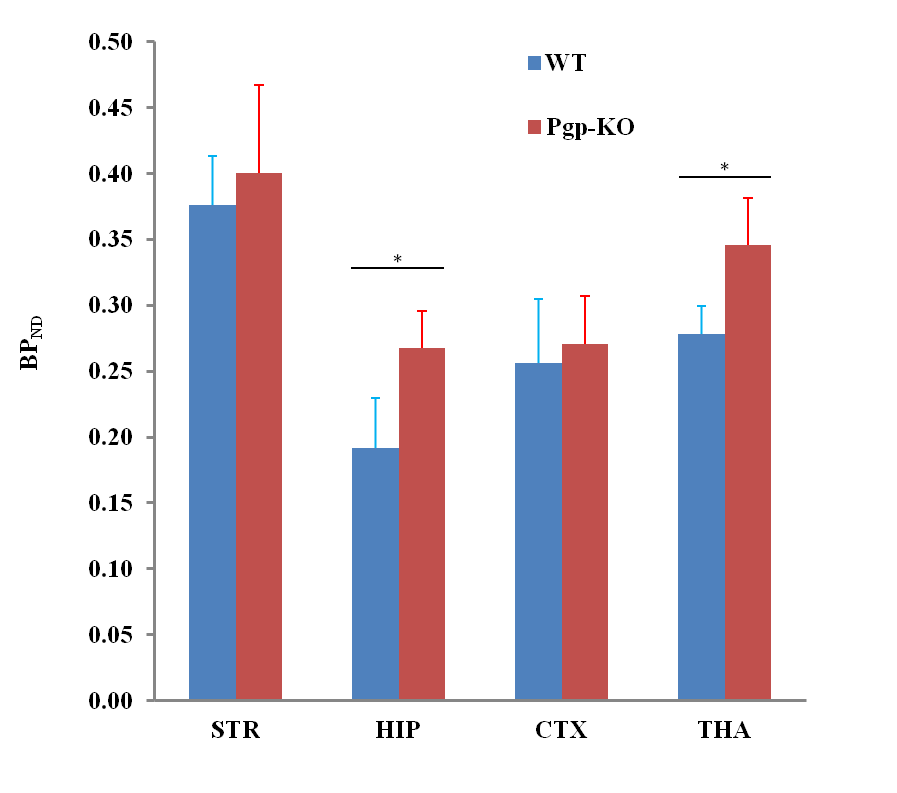

Supplement: Supplementary file 1 — Supplementary material to the manuscript by Tóth et al. Additional PET experiment using P-gp knockout and wild-type rats to explore the contribution of the ABC transporter P-gp on the brain uptake of [11C]AZ10419369. [file 13550_2014_64_MOESM1_ESM.docx]
